# Predicting the Global Distribution of *Nitraria* L. Under Climate Change Based on Optimized MaxEnt Modeling

**DOI:** 10.3390/plants14010067

**Published:** 2024-12-28

**Authors:** Ke Lu, Mili Liu, Qi Feng, Wei Liu, Meng Zhu, Yizhong Duan

**Affiliations:** 1 Shaanxi Key Laboratory of Ecological Restoration in Northern Shaanxi Mining Area, College of Life Science, Yulin University, Yulin 719000, China; luke@yulinu.edu.cn (K.L.); liumili@stumail.nwu.edu.cn (M.L.); 2 Key Laboratory of Ecological Safety and Sustainable Development in Arid Lands, Northwest Institute of Eco-Environment and Resources, Chinese Academy of Sciences, Lanzhou 730000, China; weiliu@lzb.ac.cn (W.L.); zhumeng@lzb.ac.cn (M.Z.)

**Keywords:** climate change scenario, optimized MaxEnt model, niche modeling, *Nitraria* L., species distribution models

## Abstract

The genus of *Nitraria* L. are Tertiary-relict desert sand-fixing plants, which are an important forage and agricultural product, as well as an important source of medicinal and woody vegetable oil. In order to provide a theoretical basis for better protection and utilization of species in the *Nitraria* L., this study collected global distribution information within the *Nitraria* L., along with data on 29 environmental and climatic factors. The Maximum Entropy (MaxEnt) model was used to simulate the globally suitable distribution areas for *Nitraria* L. The results showed that the mean AUC value was 0.897, the TSS average value was 0.913, and the model prediction results were excellent. UV-B seasonality (UVB-2), UV-B of the lowest month (UVB-4), precipitation of the warmest quarter (bio18), the DEM (Digital Elevation Model), and annual precipitation (bio12) were the key variables affecting the distribution area of *Nitraria* L, with contributions of 54.4%, 11.1%, 8.3%, 7.4%, and 4.1%, respectively. The *Nitraria* L. plants are currently found mainly in Central Asia, North Africa, the neighboring Middle East, and parts of southern Australia and Siberia. In future scenarios, except for a small expansion of the 2030s scenario model *Nitraria* L., the potential suitable distribution areas showed a decreasing trend. The contraction area is mainly concentrated in South Asia, such as Afghanistan and Pakistan, North Africa, Libya, as well as in areas of low suitability in northern Australia, where there was also significant shrinkage. The areas of expansion are mainly concentrated in the Qinghai–Tibet Plateau to the Iranian plateau, and the Sahara Desert is also partly expanded. With rising Greenhouse gas concentrations, habitat fragmentation is becoming more severe. Center-of-mass migration results also suggest that the potential suitable area of *Nitraria* L. will shift northwestward in the future. This study can provide a theoretical basis for determining the scope of *Nitraria* L. habitat protection, population restoration, resource management and industrial development in local areas.

## 1. Introduction

Global climate change directly affects the habitats of species, such as ecosystem structure and function, spring phenology of plants, and hydrothermal environments, which in turn profoundly affects the geographic distribution patterns of plants and may even contribute to the drastic reduction in some biodiversity [1,2]. The Sixth Assessment Report of the United Nations Intergovernmental Panel on Climate Change (IPCC 6) clearly points out that the global surface temperature during 2011–2020 has increased by 1.1 °C compared with that during 1850–1900, and the temperature will continue to rise [3]. With climate change, plants will respond to environmental changes by migrating to more suitable areas, and the geographical distribution pattern of plants will also change [4]. Many scholars believe that hydrothermal conditions are the key factors affecting plant distribution in arid areas [5]. In the context of global climate change, the survival of plants in arid areas is seriously threatened [6]. Understanding the effects of climate change on the geographical distribution pattern of plants can lay a foundation for species conservation in arid areas [7].

Ecological Niche Models (ENMs) are a type of model that predicts whether a species can survive in a region based on environmental factors and the known actual distribution of the species through specific algorithms, which can predict the potential distribution of the species in the case of global climate change and is conducive to the protection of endangered plants [8,9]. Ecological niche models have been extensively employed to investigate the historical geographical distribution of species and their prospective distribution trends [10]. The presently prevalent niche models encompass the generalized linear model (GLM) [11], generalized additive model (GAM) [12], genetic algorithm rule set generation model (GARP) [13], climate matched extreme value model (Climex) [14], maximum entropy model (MaxEnt) [15], Bioclim model [16], and Domain model [17]. Owing to their substantial computational complexity, GLM and GAM models are typically appropriate merely for data sets with well-defined probability distributions, and their predictive capabilities are relatively feeble [11,12]. The pronounced sensitivity of GARP and Climex models to the input data may give rise to result biases, especially when the data are scarce [13]. In contrast, Bioclim and Domain models predominantly rely on climate data and species location data, yet they inadequately account for other potential influencing factors [16,17]. Nevertheless, the MaxEnt model can attain a high level of simulation accuracy even with a restricted sample size, thereby garnering significant favor among numerous scholars [15]. MaxEnt models are often combined with ArcGIS and play an important role in predicting changes in endangered species habitat. For example, the prediction of potential distribution areas and priority protected areas of *Prunus mongolica* Maxim and *Ammopiptanthus mongolicus* Maxim [18,19].

*Nitraria* L. belongs to the family of Zygophyllceae, is a Tertiary relict plant of the ancient Mediterranean, and it is a dominant species in desert plant clusters from the Mediterranean to Central Asia. *Nitraria* L. plants have the characteristics of salt tolerance, poor soil tolerance, drought tolerance, and high temperature tolerance [20]. *Nitraria* L. plants have strong sand fixation and sand blocking abilities and are excellent forage and oil plants, their fruits are not only sweet and sour, but also edible, and can be used for brewing wine and vinegar [21]. *Nitraria* L. are also good medicinal materials and are known as “desert cherries” with high ecological and economic value [22,23,24]. There are 12 species in this genus, distributed in Asia, Europe, Africa, and Australia [25]. They include *Nitraria pamirica* L.I.Vassiljeva, *Nitraria praevisa* Bobrov, *Nitraria roborowskii* Kom., *Nitraria sibirica* Pall., *Nitraria sphaerocarpa* Maxim., *Nitraria tangutorum* Bobrov, *Nitraria schoberi* L., *Nitraria senegalensis*, *Nitraria billardieri* DC., *Nitrari retusa* (Forssk.) Asch., *Nitraria komarovii* Iljin and Lava and *Nitraria tridentate* Desf [22]. The genus has biological and ecological characteristics such as salinity tolerance, barrenness tolerance, drought resistance, heat tolerance, sand fixation, and soil modification and adaptability [26]. At present, the research on *Nitraria* L. mainly focuses on the classification, pollen morphology, biochemical composition, nutrient composition, biological characteristics, and exploitation value [21,27,28]. However, there is a relative lack of research on the prediction of the global geographic distribution and migration routes of *Nitraria* L. at home and abroad, and only the geographic distribution and environmental adaptation of *Nitraria* L. in the arid region of Northwest China have been studied [28]. Therefore, it is necessary to use the MaxEnt model to predict the potential global distribution and migration routes of *Nitraria* L.

In this study, ArcGIS 10.4 software and the MaxEnt model were used to predict the impact of climate change on the potential suitable distribution of *Nitraria* L., and to reveal the spatial pattern changes in *Nitraria* L. distribution that may be brought about by climate change by comparing the distribution of suitable habitats of *Nitraria* L. under different climate scenarios in the present and the future. The results of this study can help with understanding the challenges faced by *Nitraria* L. under climate change conditions, and provide a scientific basis for the development of management strategies and conservation plans, as well as the utilization of resources. In addition, this study explores the potential trends of distributional changes in *Nitraria* L. under different climate scenarios, which will serve as a reference for the study of other similar species. Based on known plant responses to climate change, we hypothesize that the potential distribution of *Nitraria* L. will change under future climate scenarios, with some low-suitable distribution habitats in South Asia or Australia shrinking and ranges in Asia expanding under the combined effects of increasing temperatures and changing precipitation.

## 2. Results

### 2.1. Result and Analysis

#### 2.1.1. Model Optimization and Accuracy Evaluation Results

**Figure 1:**
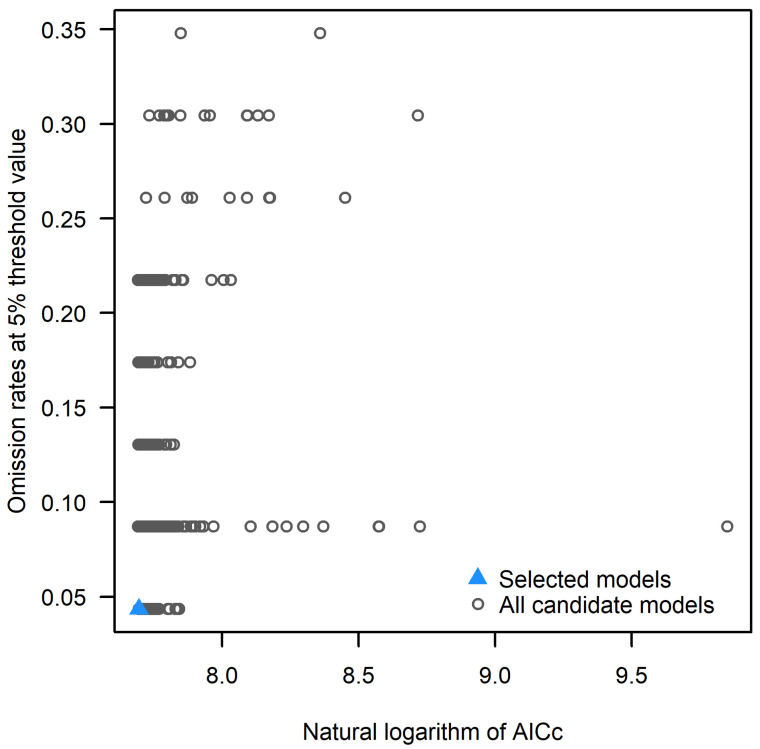
Evaluation metrics of MaxEnt model generated by ENMeval.

**Figure 2:**
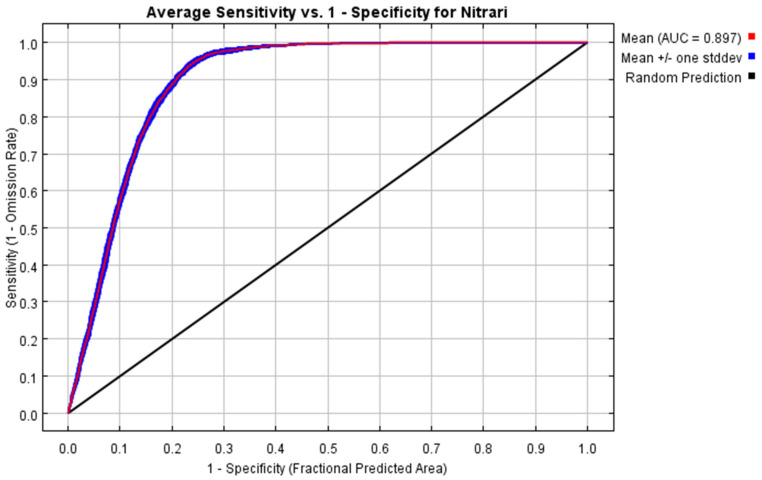
ROC curve for *Nitraria* L. using the MaxEnt model.

According to the model optimization results, FC and RM cross-checked 1240 model parameter combinations. There is one model parameter combination that meets the criteria of omission rates < 5% and smaller delta AICc, with FC choosing LQPT and an RM of 0.5. At this time, the omission rates are 0.08% and the delta AICc = 0 is small, which indicates that the prediction results of this parameter combination are better (Figure 1). The modeling results are shown in Figure 2, the average test AUC value obtained by this model is 0.897, and the mean TSS was 0.913 (Table 1), which indicates that the model has a good prediction ability.

#### 2.1.2. Important Environmental Variables Preference

**Figure 3:**
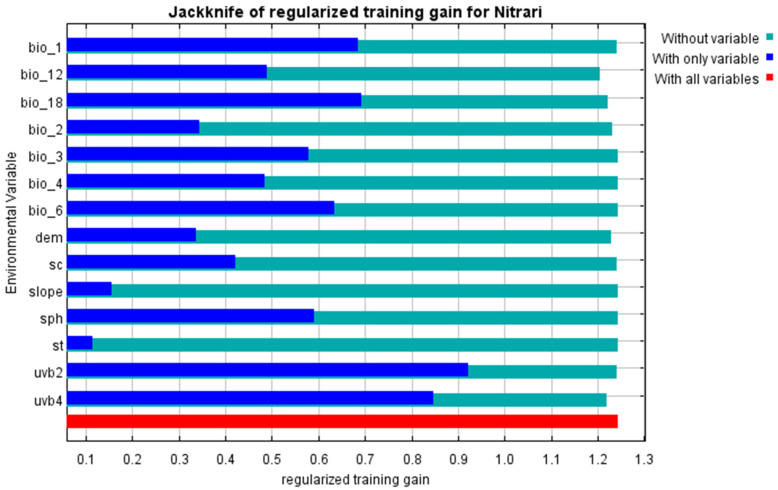
The effect of environmental variables on the distribution of *Nitraria* L. plants was evaluated by the knife-cutting method.

**Figure 4:**
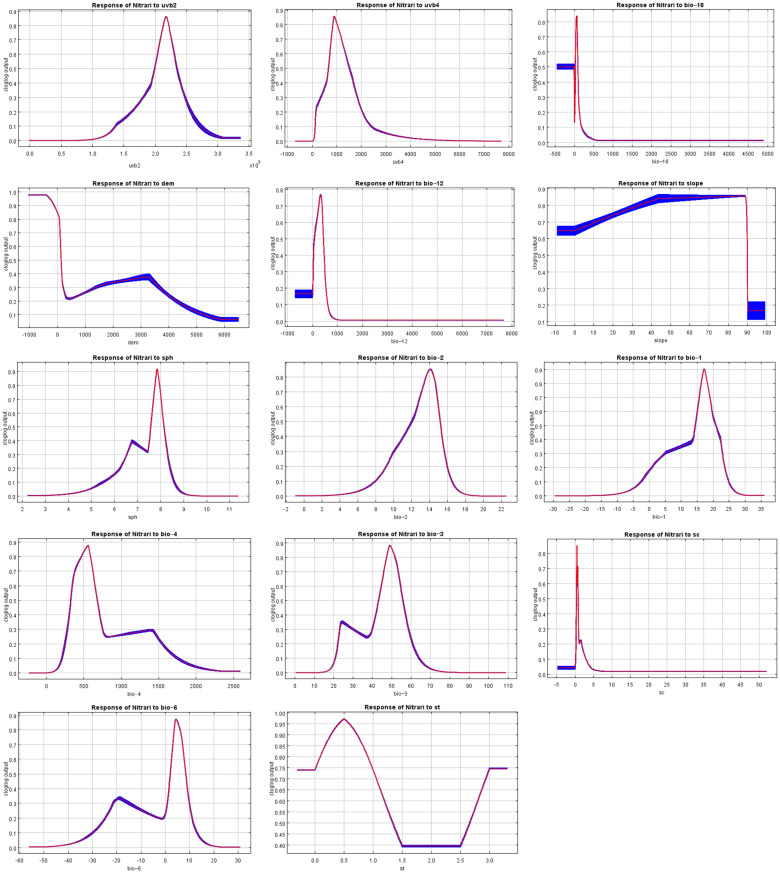
Response curves for key environmental predictors in the species distribution model for *Nitraria* L. (The red line represents the average value of all candidate models, and the blue range indicates the standard deviation, the same below).

In this study, the outputs of the MaxEnt model and the regularized training gain contribution rate, test gain rate, replacement contribution rate, and one-way response curves based on the knife-cut method revealed the dominant environmental factors affecting the geographic distribution of *Nitraria* L (Figure 3 and Figure 4; Table 2). According to Table 2, the contribution rates of mean UV-B seasonality (UVB-2), mean UV-B of the lowest month (UVB-4), precipitation of the warmest quarter (bio18), the Digital Elevation Model (DEM) and annual precipitation (bio12) are 54.4%, 11.1%, 8.3%, 7.4%, and 4.1%, respectively, with a cumulative contribution of 85.3%; the importance of replacement is 4.1%, 45.1%, 3.1%, 8.3%, and 30.1%, respectively, with a cumulative of 90.7%. This indicates that the above five environmental factors are the dominant environmental factors affecting the distribution of *Nitraria* L.

Based on the response curve, the logistics prediction for main environmental variables of *Nitraria* L. species. The suitable environmental conditions for the survival of *Nitraria* L were as follows (Figure 4, Table 2): for *Nitraria* L. UVB-2 was 2.62 × 10^4^ to 3.08 × 10^5^ J·m^−2^ day^−1^, UVB-4 was 1.83 to 6998.04 J·m^−2^ day^−1^, Bio18 was 0 to 600.7 mm, DEM was—406.0 to 5867.0 m and Bio12 was 0.0 to 1100.0 mm, respectively.

#### 2.1.3. Potential Distribution Areas Under Current Climate

**Figure 5:**
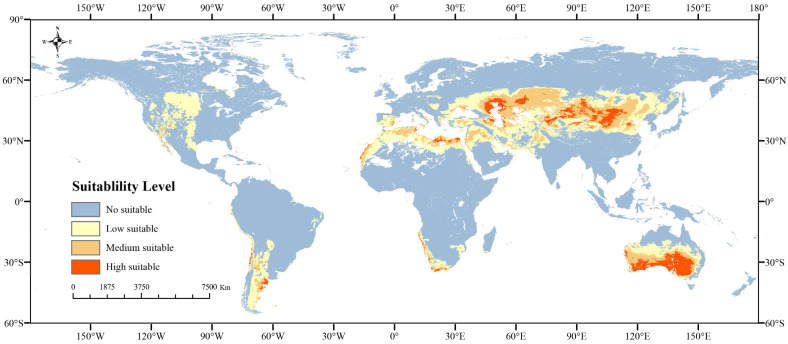
Maps of current potential habitat of *Nitraria* L. across the world.

The current total potential distribution area for *Nitraria* L. was 35.93 × 10^6^ km^2^, with a high suitable habitat accounting for 11.29% (4.34 × 10^6^ km^2^) (Figure 5, Table 3). *Nitraria* L. are widely distributed across the globe, including in Eurasia, Africa, and Australia. In Asia, *Nitraria sibirica* Pall, *Nitraria roborowskii* Kom, *Nitraria komarovii* Iljin and Lava, *Nitraria sphaerocarpa* Maxim, *Nitraria pamirica* L.I.Vassiljeva. and *Nitraria schoberi* L. are widely distributed in China in desert sands in the northwestern regions of northern Shaanxi, western Nei Mongol, Ningxia, the Gansu Hexi Corridor, Qinghai, Xinjiang, northeastern Tibet, and Mongolia. As endemic species in China, *Nitraria tangutorum* Bobrov and *Nitraria praevisa* Bobrov are found in sandy, saline lowlands and river terraces up to 3500 m above sea level. In Central Asia, for example, *Nitraria roborowskii* Kom. is found in the desert areas of Kazakhstan, Kyrgyzstan, and Uzbekistan. *Nitraria sibirica* Pall. occur in parts of Siberia. *Nitraria schoberi* L. grow widely in saline lowlands in Mongolia, Russia, Turkmenistan, Afghanistan, Iran, Saudi Arabia, and Pakistan. Not only that, *Nitraria komarovii* Iljin and Lava is also found in Russia, Turkmenistan, Iran and other countries bordering the Caspian Sea. *Nitrari retusa* (Forssk.) Asch. and *Nitraria senegalensis* Lam are endemic to Africa. *Nitrari retusa* (Forssk.) Asch. occurs mainly along the Mediterranean coast and in the northern part of the Sahara Desert. *Nitraria senegalensis* Lam occurs mainly in deserts and sparse savannas in Mauritania, Senegal, and other countries in the southwestern part of the Sahara Desert. *Nitraria tridentate* Desf is mainly found in the countries of North Africa and neighboring Arab countries. In addition, the endemic species *Nitraria billardieri* DC occurs in the savannas and deserts of southern Australia.

#### 2.1.4. Changes in the Suitable Habitat Areas of *Nitraria* L. in the Future

**Figure 6:**
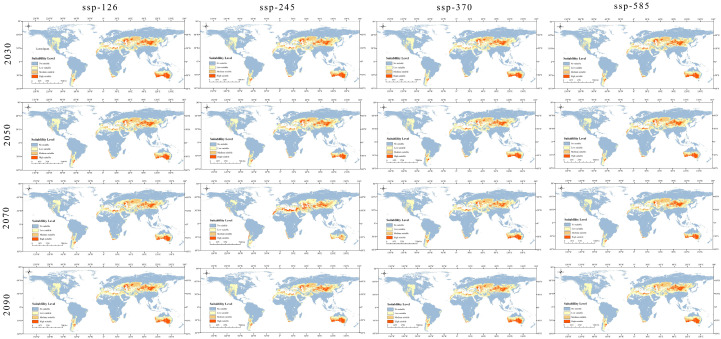
Future species distribution models (SDMs) of *Nitraria* L. under four climate change scenarios.

**Figure 7:**
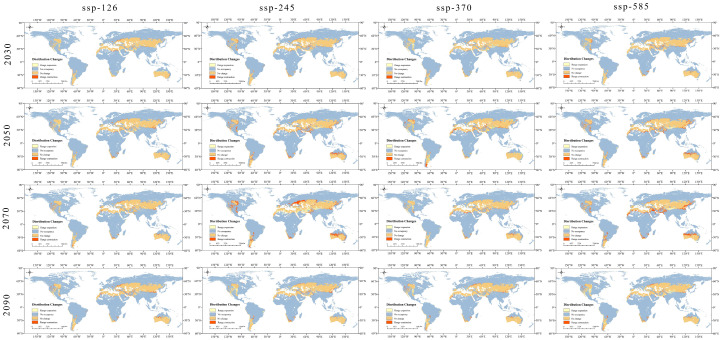
Distribution changes in the future climate scenario of *Nitraria* L. compared to the current. Red means range shrinkage, orange means range unchanged, and yellow means range expansion.

Under the four emission scenarios (SSP126, SSP245, SSP370, and SSP585) for the future period, most of the current distribution is still suitable for the distribution of *Nitraria* L., with the main ranges remaining in Central Asia, North Africa, the neighboring Middle East, and parts of southern Australia and Siberia. However, there is an increasing trend in the area occupied by highland climatic zones from the current to the future (Figure 6 and Figure 7; Table 3). The distribution of the high habitability zone has changed considerably from the current to the future, from the current distribution mainly in the central subtropical and northern subtropical regions to the expansion of the highland climatic zone, and the fragmentation is becoming gradually more serious. This is quite interesting, and it results in climatic change effects on the potential distribution of *Nitraria* L. in the future (2030s, 2050s, 2070s, and 2090s) under four climate scenarios (SSP126, SSP245, SSP370, and SSP585). The potential distribution areas of *Nitraria* L. species in the future scenarios were very similar.

In the future SSP126 scenario, the *Nitraria* L. suitable area did not change much, only in recent years to the 2030s. The potential suitable area of white spurge plants as a whole showed a trend of expansion, with the total suitable area of 36.24 × 10^6^ km^2^ and the total expansion ratio of 0.86%. The expansion of the area is mainly concentrated in the Iranian Plateau area (Figure 7). In the other three periods, the potential habitat area of *Nitraria* L. showed an overall trend of shrinkage, with a total area of 35.48–35.89 × 10^6^ km^2^, and a shrinkage ratio of 0.11%–1.25%. The shrinkage areas were mainly concentrated in the plains of the Mongolian Plain in Asia (Figure 6 and Figure 7; Table 3).

In the future SSP245 scenario, only in the recent period from the current to 2030s, the potential habitat area of *Nitraria* L. showed an overall trend of expansion, with a total area of 36.46 × 10^6^ km^2^ and a total expansion ratio of 1.48%. In the other three periods, the potential habitat area of *Nitraria* L. showed a trend of shrinkage, with a total area of 35.48–35.89 × 10^6^ km^2^ and a shrinkage ratio of 0.11%–1.25%. In the other three periods, the potential habitat area of white spurge was shrinking, with a total area of 30.94–35.65 × 10^6^ km^2^, and the proportion of shrinkage was 0.78%–13.89% (Figure 6). The contraction area is mainly concentrated in South Asia, such as Afghanistan and Pakistan, North Africa, Libya, as well as areas of low suitability in northern Australia, where there was also significant shrinkage (Figure 7). Especially in the 2070-SSP245 scenario model, a large area of contraction in the plains of eastern Europe, Romania, Morocco, Ukraine, and Siberia (Figure 7). The expansion area is mainly concentrated in Asia, for example, the Qinghai–Tibet Plateau in China to the higher elevation area of the Iranian plateau, where the nearby desert area has also expanded (Figure 6 and Figure 7; Table 3).

In the future SSP370 scenarios, the suitable distribution area of the white spurge plants does not change much. From the current to the 2030s and 2050s, the potential suitable area of the white spurge plants shows an overall trend of expansion, the total suitable area is 36.09–36.36 × 10^6^ km^2^, the total expansion ratio is 0.45%–1.53%. The expansion area is mainly concentrated in the Iranian Plateau area and Northern Pakistan. From the current to the 2070s and 2090s, the potential habitat area of *Nitraria* L. was shrinking, with a total area of 35.36–35.53 × 10^6^ km^2^, and the proportion of shrinkage was 1.11%–1.58%. The shrinkage area was mainly concentrated in the plains of Eastern Europe and the Mongolian plains in Asia (Figure 6 and Figure 7; Table 3).

In the future SSP585 scenario, the current to the 2030s and 2090s, the potential habitat area of *Nitraria* L. plants shows an overall trend of expansion, with a total area of 36.05–37.37 × 10^6^ km^2^, and a total expansion ratio of 0.33%–4.01%. The expansion area is mainly concentrated in Asia, for example, the Qinghai–Tibet Plateau in China to the higher elevation area of the Iranian plateau. In the current to the 2050s and 2070s, the potential habitable zones of *Nitraria* L. were shrinking, with a total area of 32.00–34.22 × 10^6^ km^2^, a reduction of 1.11%–1.58%. The shrinking areas were mainly concentrated in the plains of Northern Pakistan, Mongolian plains in Asia, the southern edge of the Sahara Desert, and south-central Australia. In addition, in the 2070-SSP585 scenario, these low-latitude regions located in the Mesopotamian Plain of Africa, the western Persian Gulf, the Indus Plain, and the Northeast China Plain experience substantial shrinkage, with some low-suitability areas declining or disappearing (Figure 6 and Figure 7; Table 3).

#### 2.1.5. The Spatial Shift in Potential Habitats Centroid in the Future

**Figure 8:**
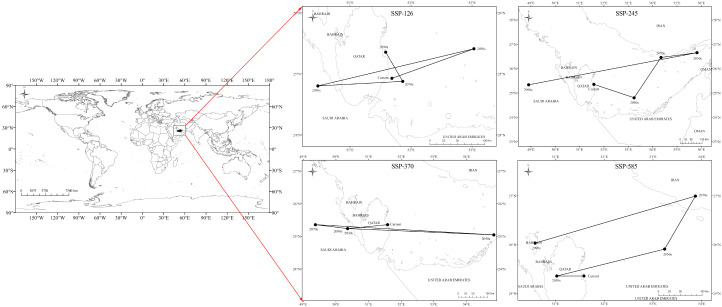
The core distributional shifts under different climate scenario/year for *Nitraria* L.

In this study, the potential habitat area of *Nitraria* L. was defined as the geometric center point to simulate the change in centroid migration under different climate scenarios. As shown in Table 4 and Figure 8, the distribution center of *Nitraria* L. was currently located in the eastern part of Qatar (51°36′45.0504″ E, 25°25′48.7632″ N). With the passage of time, in the four carbon emission scenarios, the center of gravity in the suitable distribution area gradually shifted to the northeast area (Figure 8, Table 4).

In the future SSP126 scenario, the center of mass in the 2030s shifted to the northeast by 144.526 km compared with that at the current; the center of mass in the 2050s shifted to Saudi Arabia by 133.792 km compared with that in the 2030s; the center of mass in the 2070s shifted to the northeast by 144.014 km compared with that in the 2050s; and the center of mass in the 2090s shifted to the northwest by 65.321 km compared with that in the 2070s (Figure 8, Table 4).

In the future SSP245 scenario, the center of gravity in the 2030s is 165.124 km to the southeast compared to the current one, and the center of gravity is located in the United Arab Emirates. The center of gravity for the period from the 2030s to the 2050s is located in central Bahrain, with a migration distance of 10.912 km. From the 2050s to the 2070s, the center of gravity will gradually move to the south of Iran in a northeasterly direction with a distance of 144.014 km. Instead, from the 2070s to the 2090s, the center of gravity gradually shifted toward the western Saudi Arabian region over a distance of 704.664 km (Figure 8, Table 4).

In the future SSP370 scenario, the four scenarios change slightly, and the center of gravity is located in Saudi Arabia. The centroid shift in the 2030s is 123.545 km to the south. In the 2050s, the center of mass moved 407.486 km to the east compared to the 2030s. In the 2070s, the center of mass shifted 519.618 km to the northwest compared with the 2050s. In the 2090s, the center of mass moved 112.737 km eastward compared to the 2070s (Figure 8, Table 4).

In the future SSP585 scenario, the center of gravity in the 2030s is 71.701 km to the west compared to the current one, and the center of gravity is located in Qatar. The center of gravity for the period from the 2030s to the 2050s is located in Hormozgan Province, Iran, moving 348.673 km to the northeast. From the 2050s to the 2070s, the center of gravity will gradually shift to the east of Iran at a distance of 87.125 km. However, from the 2070s to the 2090s, the center of gravity shifted 383.189 km to the southwest, to the south of Bahrain (Figure 8, Table 4).

#### 2.1.6. Conservation Status of *Nitraria* L.

**Figure 9:**
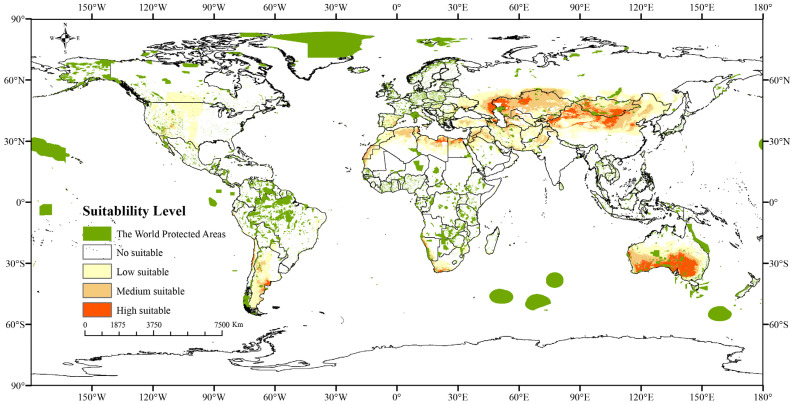
Distribution of potential suitable areas in the current protection area of *Nitraria* L.

As can be seen from Figure 9, the largest nature reserves in the world are mainly concentrated in Greenland. The main potential habitat areas of *Nitraria* L. plants in the present and future periods are still distributed in Central Asia, North Africa, Siberia, southern Australia, and the northern part of the Sahara Desert, while the nature reserves in these areas are small and heavily fragmented. Therefore, the protection of corydalis is particularly critical in Central Asia, North Africa, Siberia, southern Australia, and the northern part of the Sahara Desert. As shown in Figure 6 and Figure 9, in the scenario of simulated increasing greenhouse gasses in the future, no matter in the scenario of simulated high-concentration greenhouse gas emission or in the scenario of low-concentration greenhouse gas emission, the area of the protected area in the high suitability area changes little with the increase in greenhouse gas concentration. However, there is a decreasing trend in the size of potentially suitable areas for the genus *Nitraria* L. as greenhouse gas concentrations increase.

## 3. Discussion

As a Tertiary relict plant in the ancient Mediterranean, the *Nitraria* L. is of great ecological benefit and economic value to arid and semi-arid regions, and the study of the potential habitat and migration routes of *Nitraria* L. under climate change will be of great help to the conservation and resource utilization of this genus [22,29]. The MaxEnt model has the characteristics of predicting species distribution with a small sample size, and is widely used in the fields of suitability assessment in ecology [30,31]. In this study, we used the MaxEnt model to simulate the potential suitable area of *Nitraria* L. The higher AUC and TSS values indicated that our model had high accuracy, and the simulation results were relatively accurate, which was consistent with the actual distribution of *Nitraria* L.

### 3.1. The Influence of Environmental of Nitraria L.

In this study, the training gain value, contribution rate and single factor response curve were obtained from the optimized MaxEnt model. The main environmental factors affecting the distribution of *Nitraria* L. were annual difference in UVB-2 (UV-B seasonality), UVB-4 (mean UV-B of the lowest month), bio18 (precipitation of warmest quarter), the DEM (Digital Elevation Model) and bio12 (annual precipitation). It can be concluded that solar radiation, temperature, precipitation, and altitude are important environmental factors affecting the distribution of *Nitraria* L. within the multitude of factors influencing the habitat of *Nitraria* L. The results of the study are consistent with the fact that *Nitraria* L. plants tend to grow in arid and semi-arid regions, which are usually at higher altitudes, with thinner atmosphere and relatively strong ultraviolet radiation [28]. This study found that among many environmental impact factors, ultraviolet radiation had the greatest impact, accounting for 65.5%; this may be due to the fact that water restriction may be relatively stable in arid regions, while UV intensity varies greatly with time and weather, making the UV response of *Nitraria* L. more important in the stress response of the whole ecosystem.

Temperature and light conditions usually play a very important role in the distribution of plants [32]. Duan Yizhong et al. [29]. found that the maximum temperature and other male properties of the warmest month had a significant impact on the potential suitable areas of *Nitraria* L. in China, indicating that the temperature correlation variables had a significant effect on the distribution of *Nitraria* L. In addition, previous studies have shown that the temperature suitable for survival in northern China is around 7–9 °C, while unsuitable temperature conditions may inhibit the growth of *Nitraria* L., which was consistent with the results obtained in this study [33,34]. Solar radiation also has a direct impact on temperature changes, and studies have found that plants grow more vigorously in places with plenty of sunlight [35]. The results showed that under the current climate conditions, *Nitraria sibirica* Pall, *Nitraria roborowskii* Kom, *Nitraria komarovii* Iljin and Lava, *Nitraria sphaerocarpa* Maxim, *Nitraria pamirica* L.I.Vassiljeva., and *Nitraria schoberi* L. are widely distributed in desert and semi-desert lake basin, sandy land, river terrace, plain sandy land, and sticky land in China, Mongolia, Russia, Central Asia, and Siberia. The dry, cold, high-ultraviolet climate of these regions promotes the growth of the genus, reflecting its preference for drought and its ability to tolerate high temperatures [36]. Zhang et al. [37] found that many plants, including the *Nitraria* L., tend to survive in arid climates in a study of the distribution patterns of endemic plant groups. Our findings are consistent with theirs.

Plant survival is closely related to environmental conditions, and precipitation is also a key determinant of plant regeneration and geographic distribution [38]. The results demonstrate that, under the current climate, *Nitraria* L. plants mainly grow in Eurasia, Africa, and some desert and semi-desert areas, such as sandy areas and the Gobi Desert in Australia. These areas are dry with less rain, reflecting the ability of *Nitraria* L. plants to withstand drought and high temperatures [28]. Some scholars have found that many relict plants, such as *Tetraena* Maxim [39], *Ammopiptanthus* Maxim [19], and *Sarcozygium* Bunge [40] have a preference for surviving in dry climatic conditions. Our research findings align with their observations. Among the 14 environmental variables, the plant’s growth may be affected when the precipitation of the warmest quarter (bio18) was between 0.0 mm and 600.7 mm and the annual precipitation (bio12) was between 0.0 mm and 1100.0 mm, which was closely related to the results of Kumar et al. [41]. This study found that the plant grows well in the annual precipitation range of about 100–350 mm. This is consistent with previous empirical studies on the precipitation niche of shrub species, which suggests that such shrub species are prevalent in semi-arid climates [42,43]. Dry climates can cause water stress that limits the growth of most shrub species [44]. In areas with annual precipitation of more than 400 mm, the growth probability of *Nitraria* L. is also lower, and the humid climate may promote competition between grass communities and shrubs, thus inhibiting the development of *Nitraria* L. plants. In addition, altitude is closely related to climate change [1,35]. With an increase in altitude, the temperature decreases, the precipitation pattern changes, and the soil organic matter decreases [5,45]. In response to changes in the environment, plants change their shape, such as by growing thick and small leaves, and they will grow shorter, which is consistent with the morphological characteristics of *Nitraria* L. plants [46]. For example, as a unique species in China, *Nitraria tangutorum* Bobrov grows on sandy, saline–alkali and river terraces near lake basins at an altitude of 3500 m in northwest China [47]. *Nitraria pamirica* L. I. Vassiljeva are found in Tajikistan, near the Pamir Plateau and on the arid slopes of Xinjiang, China at an altitude of 3800–4300 m [48]. In addition, other specific environmental factors can also affect the distribution of thorns, such as *N. schoberi* L., which grows in saline lowlands and is mainly found in Mongolia, Russia, Turkmenistan, Afghanistan, Iran, Romania, and Xinjiang in China [36]. *N. komarovii* grows in the sandy areas of lake basins and is mainly found in Russia, Turkmenistan, Iran, and other countries along the Caspian Sea [49].

### 3.2. Changes in the Geographic Distribution of Nitraria L. Under Diferent Climate-Change Scenarios

Climate change has altered the structure and function of terrestrial ecosystems, therefore changing the habitats and geographic distribution of species [50]. Using the MaxEnt model, predictions were made regarding changes in the potential geographic distribution of *Nitraria* L. under current and future climate-change scenarios (2030s, 2050s, 2070s, and 2090s; SSP126, SSP245, SSP370, and SSP585; Figure 5, Figure 6 and Figure 7). The predictive discovery of the suitable habitat area of *Nitraria* L. was widely distributed in the desert or arid areas of Eurasia, Africa, and Australia in the current period, which was basically consistent with the distribution coordinates of the actual collected *Nitraria* L. plants, indicating that the model simulation effect was good. Under the four scenarios in the future, the potential suitable area of *Nitraria* L. showed a trend of gradual fragmentation, and the distribution area basically showed a decreasing trend except for a slight expansion in the 2030s scenario model. This may be due to the increase in CO_2_ emissions, overgrazing, land clearing and other human activities to exacerbate habitat fragmentation [42]. In the scenario of the low concentration greenhouse gas emissions of SSP126 in the future, the potential suitable areas of *Nitraria* L. showed a trend of contraction, except for expansion in the 2030s, which is similar to the results of Zhao Xiaofeng’s research on *Hylotelephium spectabile* [51]. Due to the increase in greenhouse gas concentration, the hydrothermal conditions in the potential suitable area of the original *Nitraria* L. plants changed. Moreover, the frequent occurrence of extreme climate aggravated the contraction of the potential suitable habitat area of *Nitraria* L. [46]. For example, the *Nitraria tridentate* Desf in North Africa and neighboring Arab countries, and the *Nitrari retusa* (Forssk.) Asch north of the Sahara Desert in Africa will both experience large-scale shrinkage., as well as *Nitraria billardieri* DC in southern Australia. In the north of the low suitability area, there is also significant shrinkage. In particular, under the 2070-SSP245 scenario, the *Nitraria sibirica* Pall. distributed in the West Siberian plain showed a large shrinkage. However, in the 2070-SSP585 scenario, these low-latitude regions located in the Mesopotamian Plain of Africa, the western Persian Gulf, the Indus Plain, and the Northeast China Plain experience substantial shrinkage, with some low-suitable areas declining or disappearing. In addition, the expansion area is mainly concentrated in Asia. For example, the distribution range of *Nitraria tangutorum* Bobrov, *N. Komarovii* and *N. schoberi* L. will expand from the Tibetan Plateau in China to the higher elevation of the Iranian plateau, and the Sahara Desert will also partly expand. Chen Chenghao et al. found that the potential habitat area of *Corydalis* DC. plants showed a trend of expanding to the high-latitude plateau climate area [52]. This study is the same as his research results, which may be due to the increasing greenhouse gas concentration leading to the continuous warming of the climate, and the environment in the plateau climate area is more suitable for the growth of *Nitraria* L. plants. This paper also found that climate change had little effect on the region from the Tibetan Plateau to the Iranian plateau and the desert area of central Australia, which may be because the genus *Nitraria* L. is suitable for growing in high-altitude areas and desert areas. However, the suitable areas in some plain areas contracted, which may be due to the large climate change in these places, the relatively low ultraviolet light, and the change in temperature and precipitation. These climatic conditions are less conducive to the growth of *Nitraria* L., resulting in lower habitat suitability within these areas. In this study, in the future simulation of increasing greenhouse gas (GHGs), the change in the area of the *Nitraria* L located within the protected area with increasing GHG concentrations is small, both in the simulation of the high GHG emission scenario and the low GHG emission scenario. However, the area of potentially suitable areas in the northwestern part of the China within the protected area shows an increasing trend with increasing GHG concentrations [28].

The results of centroid migration show that the centroid migration in different periods is entirely in the subtropical region; in the scenario of low-concentration emissions of SSP126, the centroid presents a migration of northeast–southwest–northeast–northwest; in the scenario of concentration emissions of SSP245, the centroid shifts to southeast–northeast–west, and the overall migration is still mainly in the northwest direction. In the scenario of the high concentration of SSP370, the centroid migrates to southwest–southeast−northwest−southeast, with little change in the overall migration trend, while in the scenario of the maximum concentration of SSP585, the centroid migrates to northwest−northeast−southwest, and the overall migration is still mainly in the northwest direction. Relevant studies have shown that under the background of climate warming, plant distribution has a trend of expanding to high latitudes and high altitudes [31], which is consistent with the results of this study.

In the SSP126 low-emission scenario, the center of mass migration in the suitable area was reversed, and the area where the center of mass migration was reversed was located in Qatar, where the topography of the area provided a refuge for *Nitraria* L. in the low-emission scenario (SSP126), and prevented it from migrating to higher elevations and higher latitudes. In the SSP585 scenario, climate warming made the high-altitude areas warmer and with more water, which provided the possibility of migration to higher altitude and higher latitude areas, and thus the center of mass of the potential habitat area of *Nitraria* L. continued to migrate to the northwestern high-altitude and high-latitude area under the SSP585 high-concentration greenhouse gas emission scenario [29]. Overall, the fragmentation of the habitats of *Nitraria* L. is becoming more and more serious. Whether it is the shrinking of the potential habitats under the high-concentration emission scenario or the expansion of the potential habitats under the low-concentration emission scenario, climate change has caused great impacts on the habitats of *Nitraria* L., and climate change is a serious threat to the survival of *Nitraria* L., and more *Nitraria* L. should be protected from the future climates.

### 3.3. Conservation Strategies and Recommendations

It is valuable to study the geographic distribution of species in response to climate change to provide scientific data to support species conservation. Some scholars have found that the distribution area of species formed by suitable climate change is often the “climate refuge” of species [53]. Our study reveals that the highly suitable areas for *Nitraria* L. in both contemporary and future scenarios are located in Central Asia, North Africa, the neighboring Middle East, and parts of southern Australia and Siberia. Establishing conservation areas or germplasm repositories for *Nitraria* L. in these regions could effectively protect its genetic diversity. The distribution centers of *Nitraria* L. in current and future climate scenarios are mainly located in Central Asia, North Africa, the neighboring Middle East, and parts of southern Australia and Siberia, making them key areas for *Nitraria* L. breeding. Additionally, the data indicate no records of *Nitraria* L. distribution in the mountains of western North America and the Brazilian plains of South America. However, predictive results suggest that most areas in the mountains of western North America and the Brazilian plains of South America will be highly suitable habitats for *Nitraria* L. under current and future climate scenarios. These areas have suitable hydrothermal conditions for the survival and distribution of *Nitraria* L., which provides conditions for its introduction and cultivation.

## 4. Materials and Methods

### 4.1. Data Collection

#### 4.1.1. Occurrence Data

**Figure 10:**
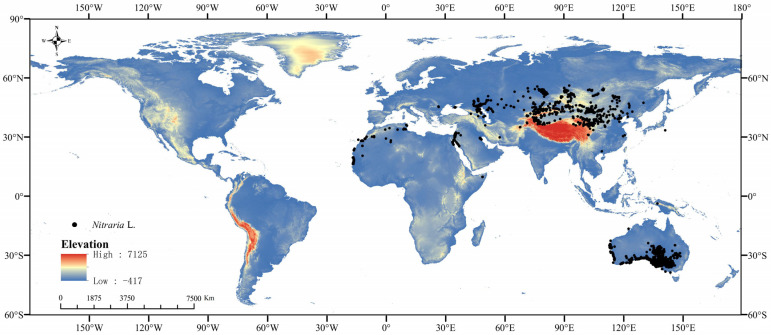
Locations of 3307 distribution points of *Nitraria* L. across the world.

We searched the distribution data 8776 of the *Nitraria* L. in the Global Biodiversity Information Network database (GBIF, http://www.gbif.org, accessed on 20 September 2024) [54]. The global distribution data of *Nitraria* L. were eliminated by ArcGIS10.4, and then the duplicates were cleaned by the “CoordinateCleaner” package in R3.6.3 to remove the data distributed in the ocean and around the capital city and the center of the country [18]. To avoid overfftting, ENMToolsv1.3 (https://www.example.com/enmtools, accessed on 20 July 2024) was used to retain only one occurrence record for each 5 × 5 km grid [55]. In the end, 5469 *Nitraria* L. occurrence records were removed and 3307 were retained (Figure 10).

#### 4.1.2. Predictor Variables

A total of 29 environmental data with a resolution of 2.5′ were obtained (Table 2), including 4 soil variables that were obtained from the Harmonized World Soil Database (HWSD, https://www.fao.org/soils-portal/en/, accessed on 17 July 2024, Table 2), 4 global UVB radiation (UVB1-4) variables that were obtained from the gIUV database (a global UV-B radiation dataset for macroe cological studies) (http://www.ufz.de/gluv/, accessed on 17 July 2024, Table 2), and 19 bioclimatic variables and 3 topographic factors that were obtained from the WorldClim2.1 (accessible at http://www.worldclim.org/, accessed on 17 July 2024) [54]. The currently available climate data span from the 1970s and 2000s. Future climate data were adopted from the Sixth Coupled Model Intercomparison Project (CMIP6), including four socio-economic pathways, namely SSP126, SSP245, SSP370, and SSP585. These pathways represent the lowest, moderate, high, and maximum emission scenarios for greenhouse gas emissions, respectively [56]. The future climate data were derived from four time periods: which are 2021–2040 (2030s), 2041–2060 (2050s), 2061–2080 (2070s) and 2081–2100 (2090s). Future data utilized the second-generation National Climate Center’s Medium-Resolution Climate System Model (BCC-CSM2-MR).

### 4.2. Data Processing and Selection

#### 4.2.1. Bioclimatic Variables Screening

**Figure 11:**
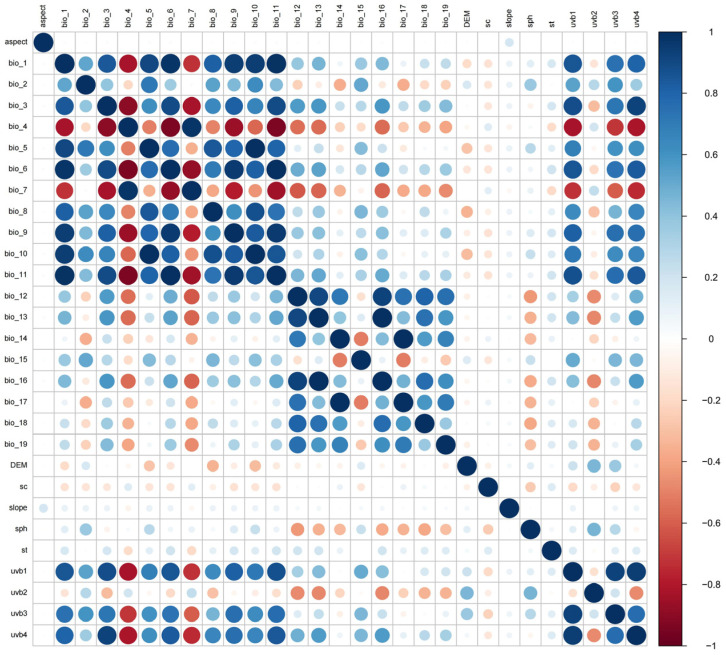
Heat map of correlation between 29 environmental variables.

In order to avoid the over-fitting of the model caused by multicollinearity between environmental data [57], a Spearman rank correlation analysis was conducted to examine the interrelationships among variables using R 3.6.3 (Figure 11). The contribution analysis of 29 variables were determined using the Jackknife test method in MaxEnt 3.4.4 software [58]. Finally, for each pair of correlated variables (r > |0.7|), only one variable with a large contribution was retained [52]. A total of 14 environmental data that were statistically and biologically significant were finally selected for modeling (Table 2).

#### 4.2.2. MaxEnt Model Optimization

The default parameters of the MaxEnt model were set by its developers based on the comparison of their model results with real distributions in their studies of bird, mammal, reptile, and plant distributions [58]. However, because the MaxEnt model is a complex model in machine learning, it is sensitive to sampling bias, and the complexity of the model is related to the feature combination (FC): LAPTH (L, linear, T, the state-valued threshold, Q, quadratic, H, fragmented hinge, P, product threshold) and regularization multiplier (RM) are significantly correlated. In order to avoid the effect of overfitting on the migration ability of the model, we used the “Kuenm” package in R3.6.3 to cross-combine the model RM (0–4) with FC (LQPTH) in 0.1 upward increments from 0 to 4, and finally choose the combination that has a small delta AICc (the difference between the calibrated optimal model and the current model’s Akaike informativeness criterion) as the optimal parameter combination to run the final model [59,60].

#### 4.2.3. Species Distribution Model Parameter Setting

The remaining parameters of the MaxEnt 3.4.4 model are set as follows: Output format is set to Cloglog, random seed is checked, and the random test percentage is set to 25%. The maximum number of iterations is set to 500, and the maximum background point number is set to 10,000, and the number of repetitions is set to 10 [30]. The Receiver Operating Characteristic Curve (ROC)’s Area Under the Curve (AUC) was used to evaluate the accuracy of the model results, with an AUC value between 0 and 1, and the larger the value, the higher the model credibility. It is generally believed that the AUC value between 0.5 and 0.7 indicates that the predictive ability of the model is average, 0.7–0.9 indicates that the predictive ability of the model is good, and 0.9–1.0 indicates that the predictive ability of the model is excellent [61]. But related studies have proven that AUC is insufficient as a predictor in spatial distribution modeling [62]. Therefore, we introduce TSS (true skill statistics) as a supplement, which inherits all the advantages of Kappa statistics and has a significant correlation with AUC statistics [63]. We select maxSSS (maximum sum of sensitivity and specificity) as the threshold, and calculate the TSS average of the 10 repeated operation results of the MaxEnt model to evaluate the model performance [63].

#### 4.2.4. Prediction of Potential Suitable Habitats

Using the “Raster Analysis Tool-Reclassification” tool in AreGIS10.4, MaxEnt is added by natural breakpoint method. The results of the model output were divided into: non-potential suitability area (0–0.135), low-potential suitability area (0.135–0.306), medium-potential suitability area (0.306–0.477), and high-potential suitability area (0.477–0.880) [64].

#### 4.2.5. Changes in the Area and Shifts in the Distribution Center of Suitable Habitats

The “Distribution Change Between Binary SDMs” tool in SDMTools was used to calculate the changes in potential habitat zones of *Nitraria* L. in different periods, and the values were defined as 0 for unfavorable, −1 for expanding, 1 for stabilizing, and 2 for contracting. In order to illustrate the temporal and spatial evolution route of the species in *Nitraria* L., we have performed the analyze the displacement of the geometric center of the potential distribution range using Centroid Changes (Lines) tool [65].

#### 4.2.6. World Protected Area Data Acquisition

From the world’s reserve data center (https://www.protectedplanet.net/en/thematic-areas/wdpa?tab=WDPA, accessed on 16 December 2024) access to the world nature reserve distribution data [66]. The Arcgis10.4 was used to understand the changes in the scope of the protected area and the potential suitable area of *Nitraria* L. in different periods in the protected area [66].

## 5. Conclusions

Using ArcGIS software and the MaxEnt model, this study predicted the potential suitable habitats of *Nitraria* L. under sixteen different contemporary and future climate scenarios. The results indicate that the key limiting factor affecting the survival and distribution of *Nitraria* L. were UV-B, temperature, precipitation, and elevation. Among these, UV-B was the most influential environmental factor in *Nitraria* L. distribution. From the perspective of potential distribution area, *Nitraria* L. is suitable for growth and distribution in arid areas. Currently, the suitable areas of *Nitraria* L. are restricted to Central Asia, Eastern Europe, North Africa, the neighboring Middle East, and parts of southern Australia and Siberia. In the future scenarios, except for a small expansion of the 2030s scenario model *Nitraria* L., the potential suitable distribution areas showed a decreasing trend. The contraction was obvious in the 2070-245 and 2070-585 scenarios. The contraction area is mainly concentrated in South Asia, such as Afghanistan and Pakistan, North Africa, Libya, as well as areas of low suitability in northern Australia, there was also significant shrinkage. The expansion area is mainly concentrated in Asia, for example, the Qinghai–Tibet Plateau in China, to the higher elevation area of the Iranian plateau; the nearby desert area has also expanded, and the Sahara Desert has partly expanded. The results of this study may provide guidance for the cultivation, genetic diversity conservation and management of *Nitraria* L. In addition, bioclimatic factors, altitude, soil type factors, and human activities may also affect the survival and distribution of white spurge. Future research will incorporate more factors into white spurge habitat prediction to further provide a theoretical basis for white spurge conservation and scientific management.

## Figures and Tables

**Table 1:** Average TSS of the results of 10 iterations of the MaxEnt model.

Models	SP_0	SP_1	SP_2	SP_3	SP_4	SP_5	SP_6	SP_7	SP_8	SP_9	Average
TSS	0.906	0.920	0.917	0.915	0.920	0.905	0.911	0.911	0.914	0.912	0.913

**Table 2:** Environmental variables and their contributions and suitable value ranges.

Environment Variable.	Description	Unit	Contribution (%)	Importance (%)	Total Suitable Range
Bio1	Annual Mean Temperature	°C	1.80	4.00	−24.10–30.58
Bio2	Mean diurnal temperature range	°C	2.60	1.60	1.00–20.62
Bio3	Isothermality (Bio2/Bio7) (×100)	-	1.20	1.10	0.37–100.00
Bio4	Seasonal variation coefficient of temperature (standard deviation × 100)	-	1.30	0.70	0.00–2350.52
Bio5	Max Temperature of Warmest Month		×	×	×
Bio6	Min Temperature of Coldest Month	°C	0.50	1.60	−40.03–23.53
Bio7	Temperature Annual Range	°C	×	×	×
Bio8	Mean temperature of the wettest quarter	°C	×	×	×
Bio9	Mean Temperature of Driest Quarter	°C	×	×	×
Bio10	Mean Temperature of Warmest Quarter	°C	×	×	×
Bio11	Mean Temperature of Coldest Quarter	°C	×	×	×
Bio12	Annual Precipitation	mm	4.10	30.10	0.00–6964.00
Bio13	Precipitation of Wettest Month	mm	×	×	×
Bio14	Precipitation of Driest Month	mm	×	×	×
Bio15	Precipitation Seasonality	mm	×	×	×
Bio16	Precipitation of Wettest Quarter	mm	×	×	×
Bio17	Precipitation of the Driest Quarter	mm	×	×	×
Bio18	Precipitation of Warmest Quarter	mm	8.30	3.10	0.00–4443.00
Bio19	Precipitation of Coldest Quarter	mm	×	×	×
SC	Soil Organic Carbon	g/kg	0.50	0.30	0.00–47.24
SpH	Soil pH	-	3.30	0.10	3.00–10.60
ST	Soil Texture	-	0.10	0.10	0.00–3.00
UVB1	Annual Mean UV-B	J/m^2^/day	×	×	×
UVB2	UV-B Seasonality	J/m^2^/day	54.40	4.10	26,212.04–307,841.00
UVB3	Mean UV-B of lightest Month	J/m^2^/day	×	×	×
UVB4	Mean UV-B of Lowest Month	J/m^2^/day	11.10	45.10	1.83–6998.04
DEM	Digital Elevation Model	m	7.40	8.30	−406.00–5867.00
Aspect	Aspect	-	×	×	×
Slope	Slope	°	3.30	0.00	0.00–47.24

Note: The variables isothermality (bio3), temperature seasonality (bio4), soil pH (SpH), soil texture (ST), and aspect are expressed as dimensionless indices or percentages. Given their nature as ratios or standardized scales, they are presented. without physical units in this table. Note: Variables without any values (indicated by ×) were removed because of high cross-correlations.

**Table 3:** Portions of different classes of potential distribution area of *Nitraria* L. under current and future climate scenarios/years.

Species	Period		Poorly Suitable Area	Moderately Suitable Area	Highly Suitable Area	Total Suitable Area
Area of each suitable area × 10^6^ km^2^ (change in the area compared to current)						
*Nitraria* L.	Current	-	20.67	10.92	4.34	35.93
	SSP1.26	2030	20.26 (−1.98%)	11.15 (2.09%)	4.83 (11.29%)	36.24 (0.86%)
		2050	20.26 (−1.98%)	11.11 (1.76%)	4.40 (1.31%)	35.77 (−0.45%)
		2070	20.39 (−1.38%)	10.89 (−0.27%)	4.20 (−3.11%)	35.48 (−1.25%)
		2090	21.00 (−10.18%)	10.42 (6.24%)	4.47 (6.31%)	35.89 (−0.11%)
	SSP2.45	2030	20.90 (1.13%)	11.12 (1.84%)	4.44 (2.30%)	36.46 (1.48%)
		2050	19.53 (−5.51%)	10.13 (−7.21%)	4.33 (−0.11%)	33.99 (−5.40%)
		2070	18.18 (−12.03%)	8.76 (−19.75%)	4.00 (−7.88%)	30.94 (−13.89%)
		2090	20.41 (−1.28%)	10.76 (−1.40%)	4.48 (3.22%)	35.65 (−0.78%)
	SSP3.70	2030	21.39 (3.46%)	10.54 (−3.47%)	4.55 (4.94%)	36.48 (1.53%)
		2050	21.22 (2.66%)	10.43 (−4.44%)	4.44 (2.32%)	36.09 (0.45%)
		2070	20.24 (−2.07%)	11.15 (2.12%)	3.97 (−8.53%)	35.36 (−1.58%)
		2090	20.65 (−0.10%)	10.57 (−3.14%)	4.31 (−0.57%)	35.53 (−1.11%)
	SSP5.85	2030	20.25 (−2.10%)	11.18 (2.32%)	4.62 (6.05%)	36.05 (0.33%)
		2050	19.56 (−5.36%)	10.21 (−6.43%)	4.45 (2.49%)	34.22 (−4.8%)
		2070	18.19 (−11.99%)	9.71 (−11.02%)	4.10 (−5.45%)	32.00 (−10.94%)
		2090	21.77 (5.32%)	11.08 (1.50%)	4.52 (4.14%)	37.37 (4.01%)

**Table 4:** Portions of different classes of potential distribution area of *Nitraria* L. under current and future climate scenarios/years.

Species	Period	Migration Distance of Centroid Shift (m)	Latiude (°)	Longitude (°)
*Nitraria* L.	Current	-	25.430212	51.612514
	2030-SSP126	144,526	25.430126	53.050112
	2050-SSP126	133,792	24.801214	50.481415
	2070-SSP126	144,014	24.922115	51.901022
	2090-SSP126	65,321	25.451025	51.620203
	2030-SSP245	165,124	24.851002	53.121016
	2050-SSP245	10,912	25.831215	53.130212
	2070-SSP245	280,183	26.601412	55.801245
	2090-SSP245	704,664	25.301512	48.910245
	2030-SSP370	123,545	25.201011	50.411241
	2050-SSP370	407,486	25.050124	54.451023
	2070-SSP370	519,618	25.321002	49.301216
	2090-SSP370	112,737	25.201011	50.413151
	2030-SSP585	71,701	25.401021	50.900121
	2050-SSP585	348,673	27.001015	53.901412
	2070-SSP585	87,125	26.371255	54.421536
	2090-SSP585	383,189	26.051326	50.601214

## Data Availability

Data are contained within the article.
